# Specific and Polyfunctional T Cell Response Against *N*-Methyl-d-aspartate Receptor in an Autoantibody-Mediated Encephalitis Model

**DOI:** 10.3390/biomedicines12112458

**Published:** 2024-10-25

**Authors:** Léonie Lesec, Julien Serrier, Célia Seillier, Benoit Bernay, Caroline Regnauld, Jonathane Furon, Jérôme Leprince, Benjamin Lefranc, Denis Vivien, Fabian Docagne, Brigitte Le Mauff, Olivier Toutirais

**Affiliations:** 1 Normandie Univ, UNICAEN, INSERM, GIP Cyceron, Institut Blood and Brain @Caen-Normandie (BB@C), UMR-S U1237, Physiopathology and Imaging of Neurological Disorders (PhIND), 14032 Caen, France; lesecleonie@gmail.com (L.L.); serrier-j@chu-caen.fr (J.S.); celia.seillier@gmail.com (C.S.); caroline.regnauld@yahoo.fr (C.R.); furon@cyceron.fr (J.F.); vivien@cyceron.fr (D.V.); fabian.docagne@inserm.fr (F.D.); lemauff-b@chu-caen.fr (B.L.M.); 2 Department of Immunology and Histocompatibility (HLA), Caen University Hospital (CHU), 14000 Caen, France; 3 Normandie Univ, UNICAEN, US EMerode, Plateform Proteogen, 14000 Caen, France; benoit.bernay@unicaen.fr; 4 Normandie Univ, UNIROUEN, INSERM, U1239 NorDiC, Neuronal and Neuroendocrine Differentiation and Communication, 76000 Rouen, France; jerome.leprince@univ-rouen.fr; 5 Normandie Univ, UNIROUEN, INSERM, US51 HeRacLeS, PRIMACEN, 76000 Rouen, France; benjamin.lefranc@univ-rouen.fr; 6 Department of Clinical Research, Caen University Hospital, 14000 Caen, France

**Keywords:** autoimmune encephalitis, *N*-methyl-d-aspartate receptor, T cells, B cells

## Abstract

Background: Anti-*N*-Methyl-d-aspartate receptor (NMDAR) autoimmune encephalitis (NMDAR AE) is an autoimmune disease characterized by severe psychiatric and neurological symptoms. While the pathogenic role of antibodies (Abs) directed against the GluN1 subunit of NMDAR is well described in this disease, the immune mechanisms involved in the generation of the autoimmune B cell response, especially the role of T helper cells, are poorly understood. Previously, we developed a B-cell-mediated mouse model of NMDAR AE by immunization with a GluN1_359–378_ peptide that drives a series of symptoms that recapitulate AE such as anxiety behaviour and spatial memory impairment. Results: In this mouse model, we identified anti-GluN1-specific CD4^+^ but also CD8^+^ T cells in both spleen and meninges. T helper cells have a polyfunctional profile, arguing for a T and B cell crosstalk to generate anti-GluN1 pathogenic Abs. Interestingly, proteomic analysis of AE meninges showed enrichment of differentially expressed proteins in biological processes associated with B cell activation and cytokine signalling pathways. Conclusions: This study identified, for the first time, a potential contribution of T helper cells in the pathology of NMDAR AE and paved the way for the development of future tolerogenic approaches to treat relapses.

## 1. Introduction

Anti-*N*-methyl-d-aspartate receptor (NMDAR) autoimmune encephalitis (NMDAR AE) was first described in 2007 as an autoimmune disorder affecting mostly young women [1]. The disease is diagnosed in patients with psychiatric symptoms including memory loss, catatonia, hallucinations, and neurologic manifestations such as seizures and autonomic dysfunctions. The cause of this encephalitis was attributed to cerebrospinal fluid (CSF) antibodies (Abs) that recognize the subunit GluN1 of the NMDAR. Patient’sCSF-derived antibodies (Abs) induce NMDAR internalization in neuron cultures in vitro, and trigger symptoms of depression and memory deficits following intrathecal infusion in mice [2,3]. Paradoxically, while pathogenic mechanisms of anti-NMDAR Abs are well documented, upstream immunological processes that lead to the humoral response are poorly understood. Follicular helper CD4^+^ T (Tfh) cells are associated with autoAb-mediated immune diseases [4]. In NMDAR AE, somatic mutations in immunoglobulin genes and isotype switching were observed in some B cells from patients’ CSF, two features indicative of T–B cell cooperation. Moreover, a study reported antibody-secreting plasma cells, T and B cell infiltration in brain perivascular spaces of patients with anti-NMDAR AE [5].

Several disease models based on active immunization with NMDAR peptide or proteins have been developed. Jones et al. have shown that injection of NMDAR proteins composed of GluN1 and GluN2B subunits embedded in liposomes induced neurological symptoms including hyperactive locomotor activity and anxiety-like behaviour [6]. Interestingly, T and B cell infiltration was found in the parenchyma of immunized mice and T cell receptor (TCR)-deficient mice did not develop the disease following immunization with NMDAR protein, suggesting that T cells are required to induce encephalitis. We have also developed a mouse model of encephalitis by immunizing mice with GluN1_359–378_ peptide that encompasses the amino terminal domain (ATD) N368/G369 described as key residues for the binding of pathogenic Abs [7]. Mice immunized with GluN1_359–378_ peptide but not with irrelevant control peptide (GluN1_168–187_ peptide) showed symptoms linked to encephalitis including spatial memory impairment, anxiety and depression-like behaviours. Two groups published that active immunization of mice against a 30-amino-acid length peptide encompassing GluN1_168–187_ sequence resulted in memory dysfunction and anxiety [8,9].

Although CD4^+^ T cell infiltration was not detected in the hippocampus in our model, it did not rule out the contribution of Th cells in B cell response into lymphoid organs.

Here, our aim was to characterize the anti-NMDAR T cell response in our AE model. GluN1-specific T cells were identified in lymphoid organs from the periphery and in the meninges of the central nervous system. In addition, we showed that AE alters protein profile towards B cell activation and cytokine production in meninges supporting a crosstalk between encephalitogenic T and B cells in the AE model.

## 2. Materials and Methods

### 2.1. Animals

C57BL/6 mice aged 6–10 weeks old from Janvier Labs were housed in our central facility, CURB (Centre Universitaire de Ressources Biologiques, Caen University, France), at controlled temperature (21 ± 1 °C) and humidity (55 ± 10%) with food and water ad libitum. All procedures were performed according to the guidelines of the institutional ethics committee (Comité Normand d’éthique en matière d’expérimentation animale, CeNomExA). The protocol was approved by the committee in accordance with the European directive N°2013/63/UE (Project licence number #23314).

### 2.2. Active Immunization

Autoimmune response was induced in 8-week-old male C57BL/6 mice (Janvier Labs, Le Genest-Saint-Isle, France) by subcutaneous injection of 200 µg of GluN1 peptide (GluN1_359–378_) with 200 µg of Complete Freund’s Adjuvant (CFA, Sigma Aldrich, Saint-Louis, MO, USA) containing 800 µg of inactivated particles of *Mycobacterium tuberculosis* (MBT, BD Biosciences, Franklin Lakes, NJ, USA). Control animals (sham) were injected with saline mixed with CFA and MBT. The emulsion was stirred at 4 °C for 1 h 30 min then administered to regions above the shoulder and the flanks into four sites (50 µL at each injection site). All animals were intraperitoneally injected at day 0 and 2 with 200 ng of pertussis toxin (Sigma Aldrich) in 200 µL of saline.

### 2.3. Isolation of Spleen and Meninges

Immunized mice were anesthetized with isoflurane 5% (Aerrane, Baxter, Deerfield, IL, USA) and intracardially perfused with heparinized phosphate buffered saline (PBS, Sigma Aldrich) after removal of the spleen. Meninges were isolated from the brain cavity after brain extraction from below.

### 2.4. Cell Culture of Leukocytes from Spleen and Meninges

Spleens were mechanically processed and the cells were passed through a 40 µm filter. Erythrocytes were lysed using a lysis buffer (0.15 M NH_4_Cl, 9 mM HKCO_3_, 0.5 M EDTA, pH 7.4 (Stemcell Technologies, Vancouver, BC, Canada) and the splenocytes were resuspended in supplemented sterile PBS with 10% foetal bovine serum (FBS, Stemcell Technologies). Meninges were enzymatically processed in PBS containing 1% collagenase-dispase (Sigma Aldrich), 0.1% DNAse I (Worthington Biochemical, Lakewood, NJ, USA), and 1% BSA (Sigma Aldrich) at 37 °C and were passed through a 70 µm filter. Cells (10^5^) were plated in 96 well U bottom plates in 200 µL of Dulbecco’s Modified Eagle’s Medium—high glucose (DMEM, Sigma Aldrich) containing 1% penicillin-streptomycin (200 mM, Sigma Aldrich), 1% Glutamax-I 100× (Gibco, Thermo Fisher Scientific, Waltham, MA, USA), 10% FBS and 0.1% 2-mercaptoethanol (Gibco), in presence of GluN1 peptide or CLIP peptide.

### 2.5. Cell Proliferation Assay by Carbofluorescein Succinimidyl Ester (CFSE) Dilution

Cells at 1 × 10^6^/mL were incubated for 20 min at room temperature in PBS with 0.5 µM CFSE (Life Technologies, Thermo Fisher Scientific). Cells were then washed and suspended in complete DMEM and were incubated with peptides as described above.

### 2.6. Flow Cytometry

Cells were resuspended in 50 µL of staining buffer and Fc receptors were blocked for 15 min at 4 °C with 10 µg/mL of anti-CD16/CD32 antibodies (BD Biosciences). Then, cells were labelled for 30 min at 4 °C in dark with fluorochrome-conjugated monoclonal antibodies BV510 anti-mouse CD3ε, APC anti-mouse CD4, PE-Cy7 anti-mouse CD8, APC-Cy7 anti-mouse CD69, FITC anti-mouse CD44, FITC anti-mouse anti-CD40L, PE anti-mouse CD45, BV421 anti-mouse CD62L, BV510 anti-mouse CD19, FITC anti-mouse CD45R-B220, APC anti-mouse GL7, PE-Cy7 anti-mouse CD138, 7-AAD (all from BioLegend, San Diego, CA, USA), and BV421 anti-mouse CD25 (BD Biosciences). Samples were acquired on FACSverse (BD Biosciences) and data were analysed with FlowJo software (7.6.5 version, BD Biosciences).

### 2.7. Cytokine Assays

Supernatants from AE splenocytes cultured with GluN1 or CLIP peptides were collected and stored at −20 °C before cytokine assay using U-PLEX Biomarker Group 1 assays (K15069L-1) kit from Meso Scale Discovery (MSD, Rockville, MD, USA).

### 2.8. Preparation of Protein Samples for Proteomics Analysis

Meninges (pool from three different animals) from AE or Sham mice were homogenized using a RIPA buffer (50 mM Tris–HCl, pH 7.5, 150 mM sodium chloride, 0.5% sodium deoxycholate, 1% Triton X-100, 0.1% SDS, and 2 mM EDTA) containing a protease inhibitor cocktail (Thermo Fisher Scientific). Then, it was kept on ice for 30 min and centrifuged at 10,000 rpm at 4 °C for 10 min. The protein concentration was quantified using the bicinchoninic acid (BCA) assay (Thermo Fisher Scientific) method. The samples with equal protein concentration were subjected to proteomic analysis.

Five µg of each protein extract were prepared using a modified Gel-aided Sample Preparation protocol (https://www.ncbi.nlm.nih.gov/pmc/articles/PMC4409837/, accessed on 2 February 2024). Samples were digested with trypsin/Lys-C overnight at 37 °C. For nano-LC fragmentation, protein or peptide samples were first desalted and concentrated onto a µC18 Omix (Agilent Technologies, Santa Clara CA, USA) before analysis.

The chromatography step was performed on a NanoElute (Bruker Daltonics, Billerica MA, USA) ultra-high-pressure nano flow chromatography system. Approximatively 50 ng of each peptide sample were concentrated onto a C18 pepmap 100 (5 mm × 300 µm i.d.) precolumn (Thermo Scientific) and separated at 50 °C onto a reversed phase Reprosil column (25 cm × 75 μm i.d.) packed with 1.6 μm C18 coated porous silica beads (Ionopticks, Fitzroy, Australia). Mobile phases consisted of 0.1% formic acid, 99.9% water (*v*/*v*) (A), and 0.1% formic acid in 99.9% ACN (*v*/*v*) (B). The nanoflow rate was set at 300 nL/min, and the gradient profile was as follows: from 2 to 15% B within 15 min, followed by an increase to 25% B within 10 min and to 37% B within 12 min and further to 9% within 7 min and re-equilibration.

MS experiments were carried out on an TIMS-TOF pro mass spectrometer (Bruker Daltonics) with a modified nano electrospray ion source (CaptiveSpray, Bruker Daltonics). A 1400 spray voltage with a capillary temperature of 180 °C was typically employed for ionizing. MS spectra were acquired in the positive mode in the mass range from 100 to 1700 *m*/*z* and 0.75 to 1.30 1/k0 window. In the experiments described here, the mass spectrometer was operated in PASEF DIA mode with exclusion of single charged peptides. The DIA acquisition scheme consisted of 24 variable windows ranging from 300 to 1000 *m*/*z*.

### 2.9. Identification of Proteins Using MS/MS Analysis

Database searching and LFQ quantification were performed using DIA-NN (version 1.8.1) (https://www.nature.com/articles/s41592-019-0638-x, accessed on 2 February 2024). An updated UniProt Mus musculus database was used for library-free search / library generation. For RT prediction and extraction mass accuracy, we used the default parameter 0.0, which means DIA-NN performed automatic mass and RT correction. The top six fragments (ranked by their library intensities) were used for peptide identification and quantification. The FDR was set to 1% at the peptide precursor level. The variable modifications allowed were as follows: Nterm-acetylation and Oxidation (M). In addition, C-Propionoamide was set as fix modification. “Trypsin/P” was selected. Data were filtered according to an FDR of 1%. Cross-run normalisation was performed using RT-dependent.

### 2.10. Differential Gene Expression Analysis

To quantify the relative levels of protein abundance between different groups, data from DIA-NN were then analysed using DEP package from R-4-4.1 version. Briefly, proteins that are identified in 2 out of 3 replicates of at least one condition were filtered, missing data were imputed using normal distribution (from Perseus, Martinsried, Germany), and differential enrichment analysis was based on linear models and empirical Bayes statistics. A 1.2-fold increase in relative abundance and a 0.05 p-value were used to determine enriched proteins.

### 2.11. Statistical Analyses

Normality tests were performed on all samples with a Shapiro–Wilk test. If normality was assumed, we used Student’s *t*-test. When normality could not be assumed, we used a non-parametric Mann–Whitney. To compare more than two groups, ANOVA tests were performed: (1) when the post-immunization duration is a comparison factor and number of animals per groups are identical, a RM two-way ANOVA with a Sidak’s multiple comparisons test is performed; (2) when the post-immunization duration time is a comparison factor and number of animals per groups are not identical, a mixed-effects model with a Sidak’s multiple comparisons test is performed; (3) when the post-immunization duration is not a comparison factor, an ordinary two-way ANOVA with a Tukey’s multiple comparisons test is performed. Results were presented as mean ± SEM and were analysed with GraphPad Prism software (version 9.0). Sample size was indicated in the text for each group and protocol. *P*-values were given in the text for each individual experiment.

## 3. Results

### 3.1. Immunization with GluN1 Peptide Did Not Modify the Adaptive Immune Cell Repartition in Both Spleen and Meninges of NMDAR AE Mice

**Figure 1:**
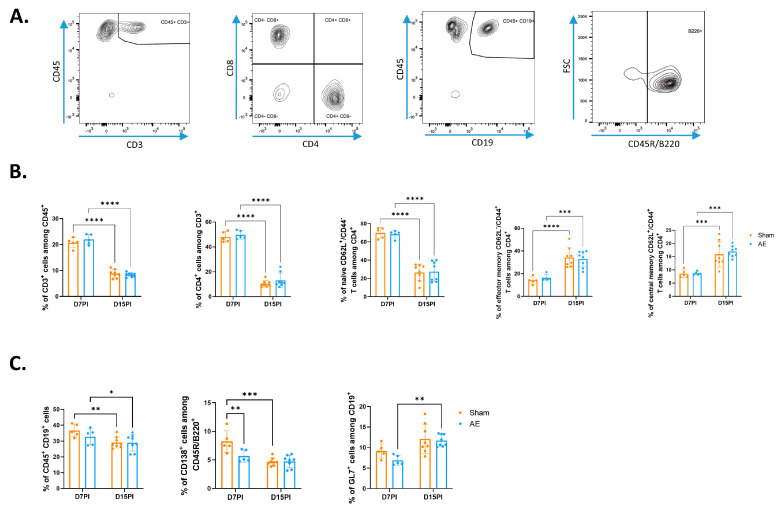
Phenotype of CD4^+^ T and B cells in spleen after immunization with GluN1 peptide. (**A**) Representative gating strategy for leukocytes in the spleen. CD3^+^ T cells and CD19^+^ B cells were gated in CD45^+^ leukocytes. (**B**) Flow cytometry analysis of T cells 7 days and 15 days post-immunization. The memory profile of CD4^+^ T cells was studied using CD44 and CD62L markers. (**C**) Flow cytometry analysis of B cells 7 days and 15 days post-immunization. The activated profile of B cells was studied using CD45/B220R and GL7 markers. Plasma cells were analysed on the basis of the expression of the CD138 marker. Data were expressed as percentages of parent cells (mean ± SEM). An ANOVA (mixed-effects model) with a Sidak’s multiple comparisons test was performed; (* *p* < 0.05; ** *p* < 0.01; *** *p* < 0.001; **** *p* < 0.0001), n = 5 (D7PI), n = 8 (D15PI). PI, post-immunization.

We analysed the distribution of the adaptive immune cells 7 days and 15 days post-immunization in spleen. At day 7, each CD4^+^ T cell subset and CD8^+^ T cell subset constituted about 40% of CD3^+^ T cells in both sham and AE mice (Figure 1A,B and Supplementary Figure S1). The CD4^+^ T cell population decreased at day 15 and represented 10% of CD3^+^ T (*p* < 0.0001) while the CD8^+^ subset increased and constituted 60% of CD3^+^ T cells (*p* < 0.0001).

At day 7 and day 15, the repartition in naive/memory CD4^+^ T cells was similar in both sham and AE mice (Figure 1B). Naive CD4^+^ T cells were the major subset at day 7 but declined at day 15, whereas the T central memory cells (Tcm, CD4^+^CD62L^+^/CD44^+^) and T effector memory cells (Tem, CD4^+^CD62L^−^/CD44^+^) subsets approximatively doubled during this interval. For CD8^+^ T cells, no modification in naive/memory phenotype was observed between day 7 and day 15 (Supplementary Figure S1).

While the frequency of CD19^+^ B cells was similar in sham or NMDAR AE mice (37% vs. 33% at day 7 respectively), we observed a weak decrease of activated GL7^+^ B cells and CD138^+^ plasma cells only 7 days post-immunization in AE mice (Figure 1C). Between day 7 and day 15, frequencies of activated GL7^+^ B cells increased in AE mice (*p* = 0.0030).

**Figure 2:**
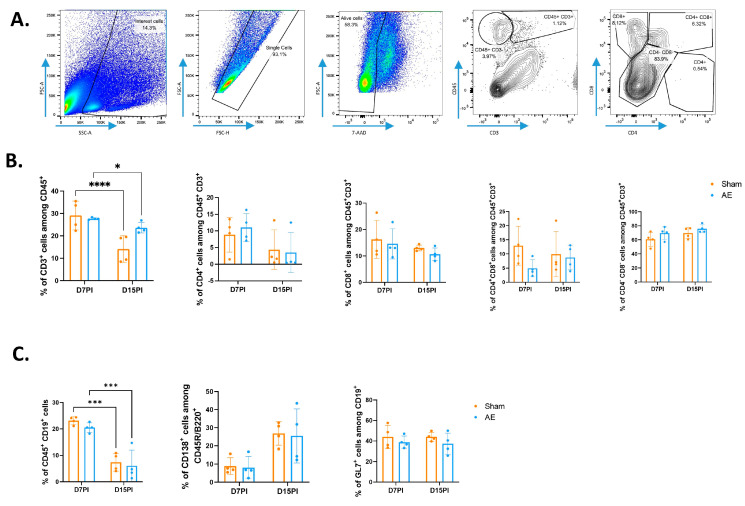
Phenotype of CD4^+^ T and B cells in meninges after immunization with GluN1 peptide. (**A**) Representative gating strategy for leukocytes in the meninges. CD3^+^ T cells were gated in viable (7-AAD negative) CD45^+^ leukocytes. (**B**) Flow cytometry analysis of T cells 7 days and 15 days post-immunization. (**C**) Flow cytometry analysis of B cells 7 days and 15 days post-immunization. The activated profile of B cells was studied using CD45/B220R and GL7 markers. Plasma cells were analysed on the basis of the expression of the CD138 marker. Each sample represents a pool of three different animals. Data were expressed as percentages of parent cells (mean ± SEM). A RM two-way ANOVA with a Sidak’s multiple comparisons test was performed (* *p* < 0.05; *** *p* < 0.001; **** *p* < 0.0001), n = 4. PI, post-immunization.

At day 7, meninges of sham or AE mice contained about 20% of CD3^+^ T cells among leukocytes (21% vs. 20%, respectively) with a small proportion of CD4^+^ T cells (10% vs. 12.3% respectively) and a majority of CD8^+^ T cells (41% vs. 30.3% respectively) (Figure 2A,B). At day 15, the percentages of CD3^+^ T cells and CD4^+^ were similar, whereas CD8^+^ T cells were less abundant in sham or AE mice (*p* = 0.0556 for AE mice and *p* = 0.0034 for sham mice). Seven days following immunization, the percentage of CD19^+^ B cells was 20% of leukocytes in meninges from sham or AE mice (Figure 2C). The percentages of activated GL7^+^ B cells (44% vs. 39% at day 7 for sham vs. AE conditions respectively) and CD138^+^ plasma cells (9% vs. 8% at day 7 for sham vs. AE conditions respectively) were not modified in both situations. At day 15, the proportion of CD19^+^ B cells significantly decreased compared to day 7, despite no significant change in activated GL7^+^ B cells or CD138^+^ plasma cells.

### 3.2. An Anti-GluN1-Specific T Cell Response Is Elicited Both in Periphery and in CNS of NMDAR AE Mice

Following a 2-day ex vivo reactivation of T cells with the GluN1 peptide, we identified specific CD4^+^ T cells among isolated splenocytes, based on the expression of the CD69 early activation marker mainly at day 15. Both the frequency of CD4^+^/CD69^+^ cells and MFI increased after reactivation: 8% ± 1.12 and a CD69 MFI of 3128 ± 223 after reactivation with GluN1 peptide, as compared to 3.68% ± 0.69 and an MFI of 1869 ± 82 with the control peptide (*p* = 0.0008 and *p* < 0.0001, respectively). The middle/late activation marker CD25 was also enhanced at day 7 and day 15 but only in terms of MFI.

We also detected GluN1-specific T cells within the CD8^+^ subset from splenocytes of AE mice at day 15, with a CD69 MFI of 3606 ± 175 with GluN1 peptide compared to 2570 ± 90 with the control peptide (*p* < 0.0001) (Supplementary Figure S2). An increase in the frequencies of CD8^+^/CD69^+^ cells and CD8^+^/CD25^+^ cells was observed after reactivation: 48.3% ± 3.2 were CD8^+^/CD69^+^ T cells and 29.9% ± 2.1 were CD8^+^/CD25^+^ T cells after GluN1 peptide reactivation, as compared to 24.3% ± 2.5 and 24.4% ± 1.6 with the control peptide, respectively (*p* < 0.0001 for CD69 and *p* = 0.0255 for CD25).

Gating on large T cells with the FSC and SSC parameters is commonly used to monitor T cell activation [10,11]. When we apply this method, the detection of GluN1-reactive T cells was enhanced (Supplementary Figure S3). We also confirmed the presence of anti-GluN1-specific CD4^+^ and CD8^+^ T cells by CFSE-based cell proliferation assay in total and large cells; there was a proliferation index of 2.33 ± 0.05 with GluN1 peptide vs. 1.12 ± 0.03 with control peptide in AE mice in large CD4 cells (*p* < 0.0001). Furthermore, a strong response of T cells to GluN1 was obtained in IFN-γ ELISPOT assay (Supplementary Figure S4).

**Figure 3:**
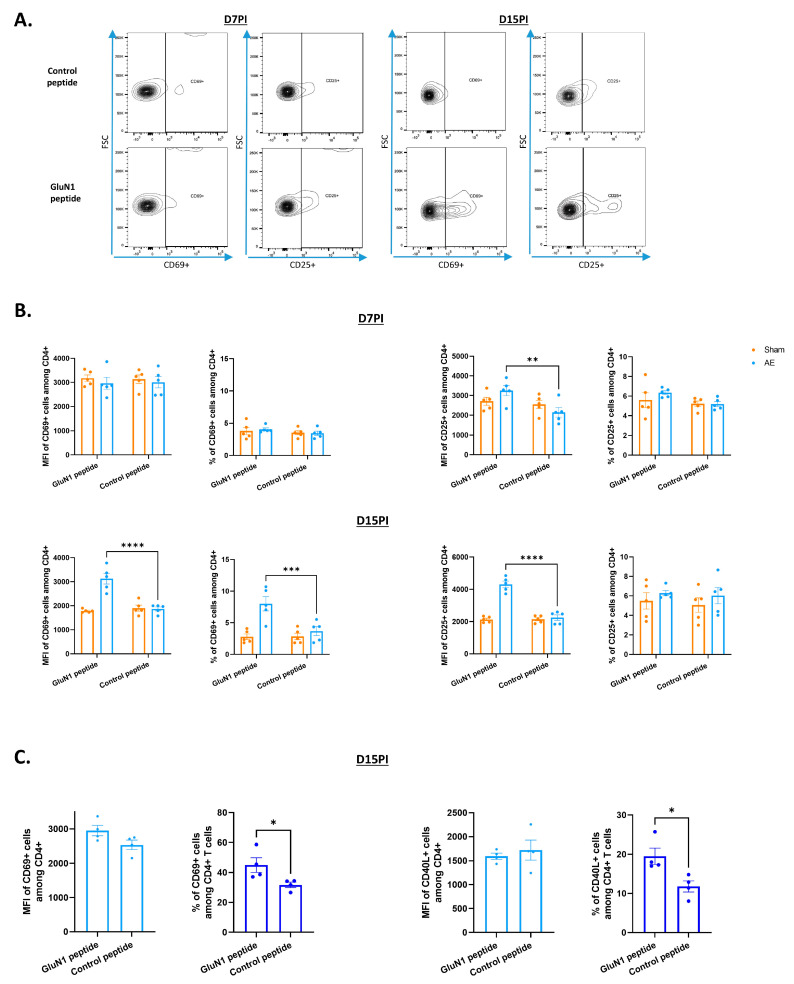
Specific CD4^+^ T cell response in the spleen and meninges of AE mice. Splenocytes or meningeal cells from sham or AE mice were cultured with GluN1 peptide or the control CLIP peptide for 2 days. (**A**) Representative gating strategy for leukocytes in the spleen. CD4^+^ T cells were gated in CD3^+^ cells. (**B**) Flow cytometry analysis of GluN1 reactive CD4 T cells in spleen at 7 days and 15 days post-immunization based on CD69 and CD25 markers. ANOVA test (** *p* < 0.01; *** *p* < 0.001; **** *p* < 0.0001), n = 5 (D7PI and D15PI). (**C**) Flow cytometry analysis of GluN1-reactive CD4^+^ T cells in meninges based on CD69 and CD40L markers, at 15 days post-immunization. Each sample represents a pool of three different animals. Data were expressed as percentages of parent cells (mean ± SEM), *t*-test test (* *p* < 0.05), n = 4.

Interestingly, CD4^+^ T cells able to recognize the GluN1 peptide were also detectable in meninges of AE mice at day 15 post-immunization: 45% ± 4.92 CD4^+^/CD69^+^ T cells and 19.5% ± 4.15 CD4^+^/CD40L^+^ T cells after reactivation with GluN1 peptide, as compared to 31.7% ± 1.8 CD4^+^/CD69^+^ T cells and 11.8% ± 2.9 CD4^+^/CD40L^+^ T cells with the control peptide (*p* = 0.0445; *p* = 0.0286, respectively) (Figure 3C). No difference in the CD69 and CD40L MFI was observed between CD4^+^ T cells from sham and EA mice.

### 3.3. Anti-GluN1-Specific T Cells Preferentially Display a Th1, Th2/Tfh, and Th17 Profile

**Figure 4:**
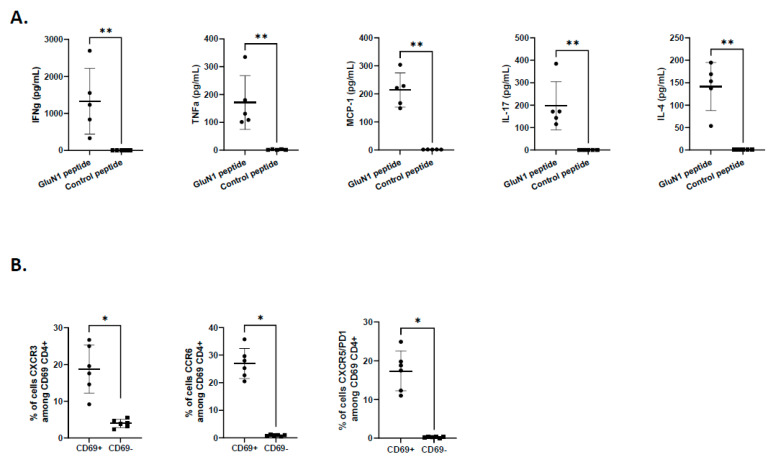
Functional profile of GluN1-specific T cells. (**A**) Splenocytes from immunized mice were cultured with GluN1 peptide or CLIP peptide for 48 h and supernatants were collected. Cytokine production was measured by ELISA (n = 5). (**B**) CD4^+^ T cells expressing the CXCR3, CCR6, and CXCR5/PD1 markers were identified among CD69^+^ or CD69^−^ cells (n = 6). Data were expressed as mean ± SD. Normality could not be assumed so a non-parametric Mann–Whitney test was used (* *p* < 0.05; ** *p* < 0.01).

Next, we analysed the functional properties of anti-GluN1-specific CD4^+^ T cells in AE mice. Following reactivation with GluN1 peptide, splenocytes exhibited a polyfunctional profile and secreted Th1 (IFN-γ, TNF-α and MCP1), Th17, and Th2/Tfh (IL-4) cytokines (Figure 4A). The analysis of the T helper profile using chemokine and inhibitory receptors corroborated this finding. Indeed, GluN1-reactive T cells significantly expressed higher levels of CXCR3, CCR6, and CXCR5/PD1 associated with Th1, Th17, and Tfh profiles, respectively (Figure 4B).

### 3.4. AE Alters Protein Profile Related to B Cell Activation and Cytokine Production in Meninges

**Figure 5:**
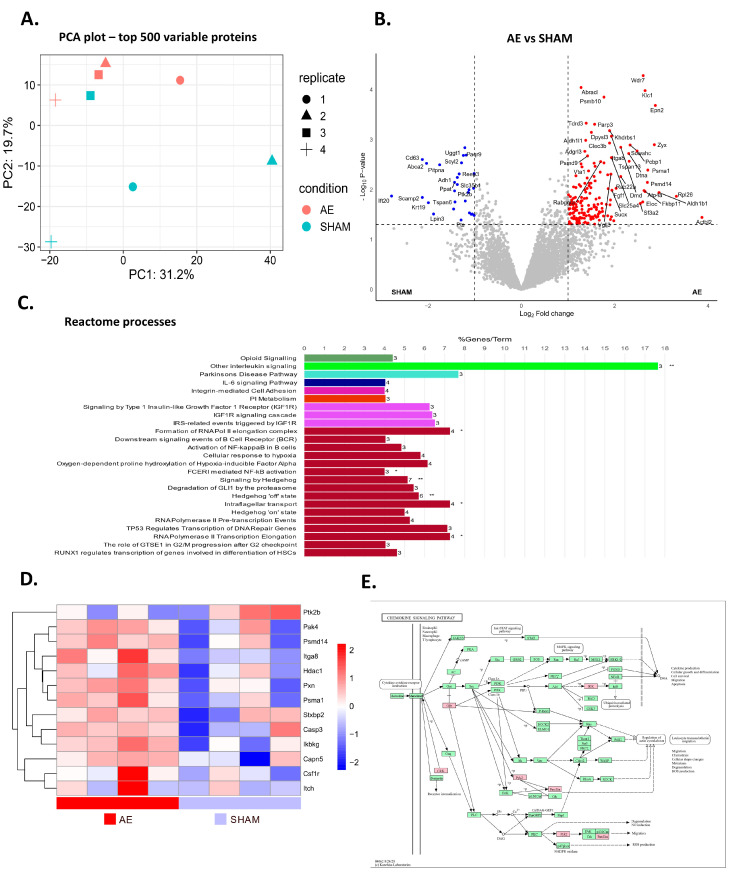
AE alters meninge protein profile in AE mice. (**A**) Principal component analysis demonstrating distinct protein profiles between AE and SHAM mice in the meninges. (**B**) Volcano plot depicting 124 upregulated and 18 downregulated proteins in AE mice relative to sham mice in meninges. (**C**) Bar graph illustrating the Reactome biological processes enriched in differentially expressed proteins (* *p* < 0.05, ** *p* < 0.01). (**D**) Heatmap showing the normalized expression of differentially expressed proteins involved in the following Reactome terms: activation of NFκB in B cells, downstream signalling events of B Cell Receptor (BCR), other interleukin signalling, IL-6 signalling, RUNX1 transcription regulation of genes involved in differentiation of HSCs, and integrin-mediated cell adhesion. (**E**) Mapping of differentially expressed proteins involved in cytokine signalling KEGG pathway (mmu04062). Each sample represents a pool of three different animals.

The analysis of differentially expressed proteins in the meninges of AE compared to sham mice revealed that they clustered separately, although a limited overlap was observed between the groups. (Figure 5A). Among all identified proteins, 124 were upregulated and 18 were downregulated in AE mice compared to sham mice (Figure 5B). In addition, reactome analysis showed enrichment of differentially expressed proteins in processes associated with B cell activation and cytokine signalling. Reactome terms included activation of NFκB in B cells, downstream signalling events of B Cell Receptor (BCR), other interleukin signalling, IL-6 signalling, RUNX1 transcription regulation of genes involved in differentiation of hematopoietic stem cells (HSCs), and integrin-mediated cell adhesion. Analysis of proteins associated with these processes revealed upregulation of 12 proteins in AE mice. Ptk2B was the only protein exhibiting a downregulation in AE mice (Figure 5D). Moreover, mapping of differentially expressed proteins involved in cytokine signalling KEGG pathway (mmu04062) highlighted their potential role in cytokine production, cellular growth and differentiation, cell survival, migration, and apoptosis, mediated in part through NFκB pathway activation via Ikbkg (IKK), and leukocyte transendothelial migration mediated by paxillin (PXN) (Figure 5E). These data suggest a distinct protein profile in the meninges of AE mice compared to sham counterparts, indicating heightened activation of B cells and alterations in their secretory activity, likely through enhanced cytokine production.

## 4. Discussion

Accumulating evidences exist about the pathogenic roles of anti-NMDAR Abs that disturb the glutamatergic signalling on neurons by mainly inducing NMDAR internalization [12]. However, the immune mechanisms that lead to anti-NMDAR Ab production remain poorly explored. Here, we examined the effects of the immunization with GluN1 peptide on T and B cell compartments in the spleen and meninges of AE mice. As compared to the sham condition, immunization with the specific peptide did not induce marked modifications in the global frequencies of B and T lymphocyte subsets. Nevertheless, in both conditions, we observed some changes at the two time points after immunization. In the spleen, the frequency of CD8^+^ T cells was increased at the expense of the CD4^+^ T cell subset at day 15 post-immunization compared to day 7. The naïve/memory profile of CD4^+^ but not CD8^+^ T cells was modified and directed towards effector memory CD4^+^ T cells. This observation is relevant since Lee et al. have demonstrated that bystander activated memory CD4^+^ T cells have a pathogenic function in EAE [13]. Unexpectedly, the frequency of B cells was decreased in the meninges at day 15 compared to day 7. These modifications that were independent of the stimulation with the GluN1 peptide may be related to the immunostimulatory effects of CFA and/or pertussis toxin. Interestingly, we found a subset of double-negative CD3^+^ T cells in the meninges. This subset represents unconventional T cells including γδ T cells, mucosal-associated invariant T, and NK-T, and emerges as an important immune component in regulation of brain homeostasis and diseases [14]. The pathogenic role of these subsets in AE is currently unknown, but would deserve further investigation

Using several assays, we described for the first time a specific T cell response against the immunizing GluN1_359–378_ peptide that induced AE-like symptoms in our model. Anti-GluN1-specific T cells were mostly detected in the spleen 15 days after the initial immunization, consistent with data from active EAE models [15,16]. The activation markers used showed generally consistent kinetics: CD69 was increased only at day 15 post-AE for CD4^+^ and CD8^+^, in total and large cell populations, based on both percentage and MFI. CD25 expression, however, was upregulated at day 7 and day 15 post-AE for CD4^+^ T cells, but only in terms of MFI. For CD8+ T cells, CD25 was upregulated at day 15 on the percentage parameter. Under our short-term (2-day) ex vivo antigen reactivation conditions, CD69 appeared more relevant and robust to detect GluN1-specific T cells than CD25. Further experiments using tetramer staining might be useful to quantity GluN1-specific T cells and analyse the kinetics of T cell activation at earlier time points. In AE mice, the helper T cell profile as determined by cytokine assay and chemokine receptor phenotyping was not skewed but polyfunctional with Th1, Th17, and Th2/Tfh cells, as described in EAE [17,18]. These data suggest that multiple immune interactions with various cell types may be required to trigger the AE disease. Our findings corroborated data published by Jones et al. in another AE mice model induced by immunization with NMDAR embedded in liposomes [6]. Using TCRα-deficient mice, authors showed that anti-NMDAR Ab production and the trigger of behavioural symptoms required T cells. To date, only one study has investigated the anti-GluN1 peripheral CD4^+^ T cell response in AE patients. Unexpectedly, Dao et al. found a lower frequency of antigen specific CD4^+^ T cells in NMDAR AE patients than in healthy donors, suggesting that T cell help is not required for anti-NMDAR Ab production [19]. However, this study has two major limitations: first, biological analyses were performed with samples from patients collected after immunotherapy that could induce deletion or anergy of anti-GluN1-specific CD4^+^ T cells even if anti-*Candida albicans* CD4^+^ T cell response is similar in the cohorts of patients and healthy donors; secondly, authors have measured the specific production of TNF-α and IFN-γ, two Th1-associated cytokines, without showing any difference between patients and healthy donors. It is possible that anti-GluN1 CD4^+^ T cells in NMDAR AE patients concerned other Th profiles such as Th17 or Tfh profiles, and so required the study of other cytokines such as IL-17 or IL-21.

Interestingly, we also detected specific CD4^+^ T cells in meninges associated with a high frequency of activated B cells and plasma cells. Proteomic analysis in meninges also provided data in favour of B cell activation. Altogether, these results suggest a potential cross-talk between meningeal T and B cells. As described in the EAE model, activated B cells may act as antigen presenting cells and restimulate specific T cells [20,21]. Activated B cells may also promote neuroinflammation by producing pro-inflammatory cytokines such as TNF-α, GM-CSF, and IL-6 [22,23,24]. In turn, CD4^+^ T cells could potentiate B cell and plasma cell response [25,26]. For example, it was shown in a passive transfer model of EAE that Th17 cells are involved in the formation of ectopic lymphoid follicle-like structures containing T and B cells in subarachnoid space [26]. The role of brain-infiltrating Tfh cells is poorly understood in neuroinflammatory models. Guo et al. have shown a correlation between infiltration of Tfh cells in the spinal cord and EAE score, and Quinn et al. described that Tfh cells contribute to disease severity in the EAE model, induced by passive transfer of Th17 cells [27,28].

Several T helper profiles have been identified among splenocytes in our AE model. Approaches of nucleic acid or protein high resolution profiling from tissues would be more appropriate to characterize the T cell functional profile in meninges [29]. On the other hand, experiments of passive co-transfer of in vitro polarized anti-GluN1 T helper cells with anti-GluN1 Abs would be useful to decipher the immune mechanisms in NMDAR AE as it was performed in the neuromyelitis optica rodent model with the aquaporin 4 autoantigen [30].

Of note, we also detected anti-GluN1-specific CD8^+^ T cells in the spleen and meninges of AE mice. In several studies, encephalitogenic anti-myelin CD8^+^ T cells have been described in EAE [31,32]. Their mechanisms of action are not clearly established. For instance, Wagner et al. have shown that anti-myelin CD8^+^ T cells enhanced reactive oxygen species production by monocyte-derived cells in the brain of EAE mice [33]. Regulatory function of CD8^+^ CD28^−^ T cells was also reported in the EAE model. So, further investigation is needed to know the function of anti-GluN1-specific CD8^+^ T cells in AE mice.

In conclusion, we demonstrated that GluN1-reactive T cells were generated both in spleen and meninges in the NMDAR AE model, suggesting their involvement in the pathogenic B cell response. Although many challenges remain to be overcome, tolerogenic strategies targeting effector T cells may provide an efficient approach to prevent relapses in AE patients.

## Data Availability

The original contributions presented in the study are included in the article/Supplementary Material, further inquiries can be directed to the corresponding author. Proteomic data are available on the website: https://www.iprox.cn/page/home.html (number: IPX0010054001).

## Supplementary Materials

The following supporting information can be downloaded at: https://www.mdpi.com/article/10.3390/biomedicines12112458/s1, Figure S1: Phenotype of CD8^+^ T cells after immunization with GluN1 peptide; Figure S2. Specific CD8^+^ T cell response in the spleen of AE mice; Figure S3. anti-GluN1 T cell response in large cells after in vitro reactivation; Figure S4. Specificity of the anti-GluN1 T cell in AE mice.
