# Supplementary material for: Specific and Polyfunctional T Cell Response Against N-Methyl-d-aspartate Receptor in an Autoantibody-Mediated Encephalitis Model
Source: Biomedicines. 2024 Oct 25;12(11):2458. doi: 10.3390/biomedicines12112458 (PMC11591936; doi:10.3390/biomedicines12112458)
Supplement: Supplementary file 1 [file biomedicines-12-02458-s001.zip › biomedicines-3180822-supplementary.pdf]

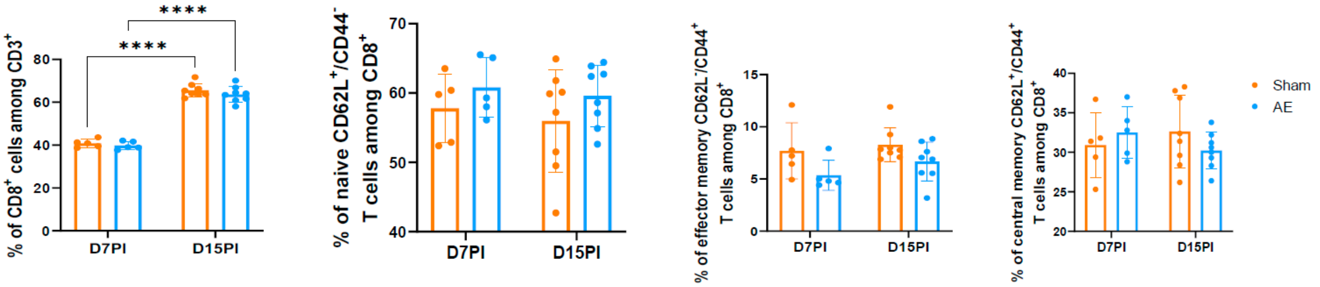

Figure S1. Phenotype of CD8⁺ T cells after immunization with GluN1 peptide. (**A, B**) Flow cytometry analysis of CD8⁺ T cells 7 and 15 days post immunization. The memory profile of CD8⁺ T cells was studied using CD44 and CD62L markers. Data were expressed as percentage of parent cells (mean ± SEM). An ANOVA (mixed-effects model) with a Sidak's multiple comparisons test was performed. (**** $p < 0.0001$), n = 5 (D7PI), n = 8 (D15PI).

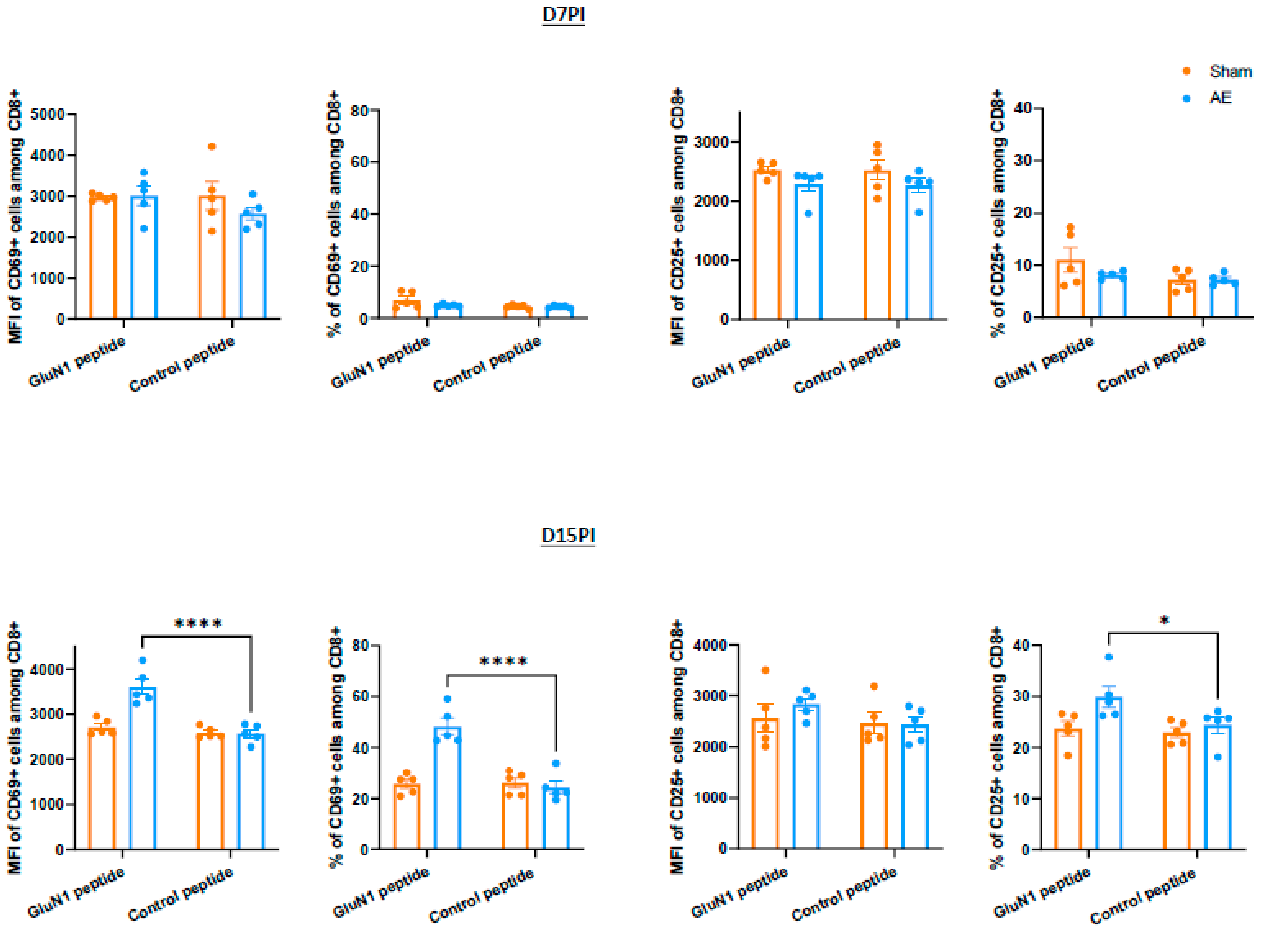

Figure S2. Specific CD8⁺ T cell response in the spleen of AE mice. Splenocytes from sham or AE mice were cultured with GluN1 peptide or the control CLIP peptide for 2 days. CD8⁺ T cells were gated in CD3⁺ cells. GluN1 reactive CD8⁺ T cells at 7 days and 15 days post immunization were identified using CD69 and CD25 markers. Mean Fluorescence Intensity (MFI) of these markers are also represented. Data were expressed as percentage of parent cells (mean ± SEM), ANOVA test (* $p < 0.05$; **** $p < 0.0001$), n = 5 (D7PI and D15PI).

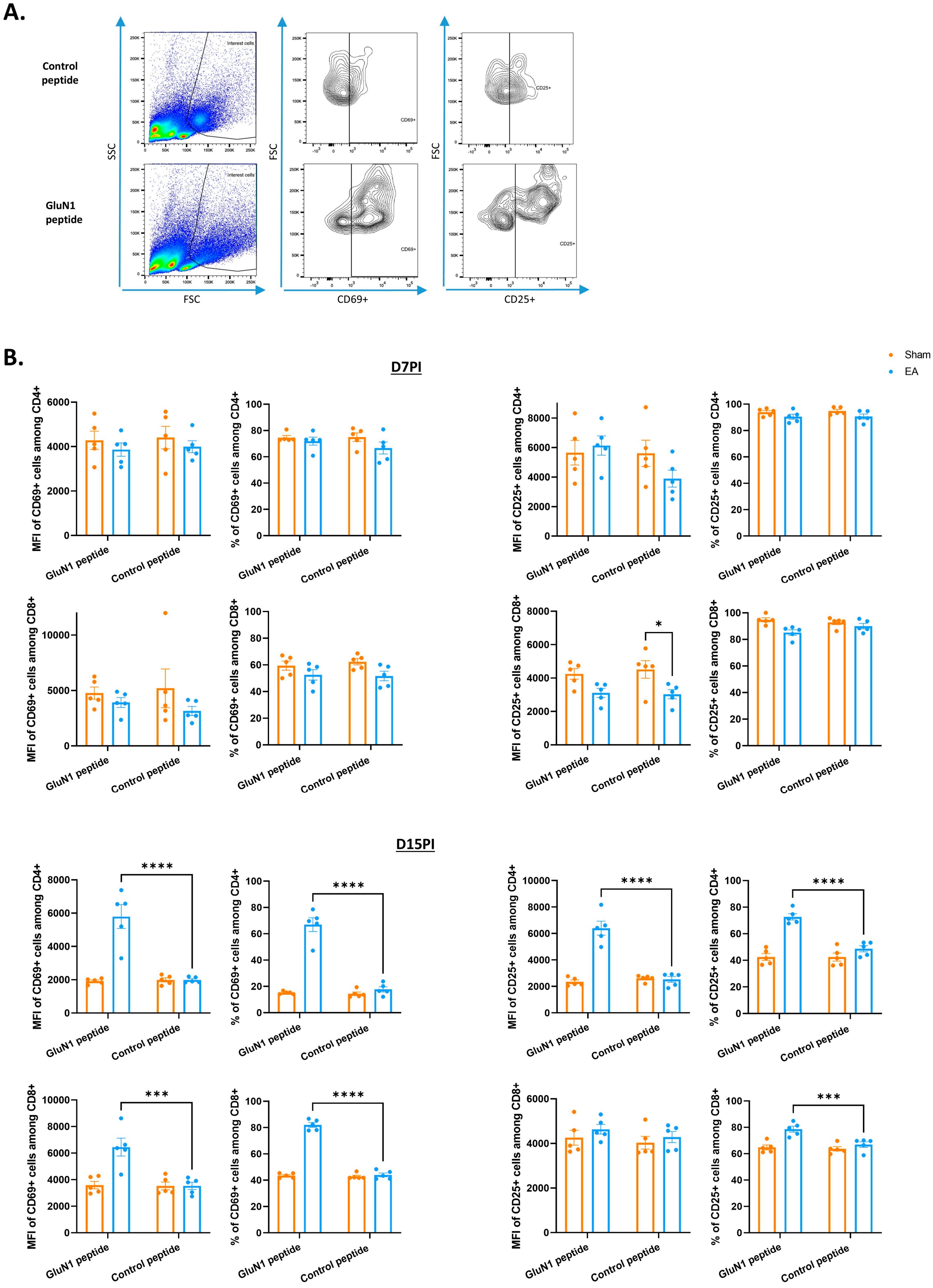

Figure S3. anti-GluN1 T cell response in large cells after *in vitro* reactivation. Splenocytes from sham or AE mice were cultured with GluN1 peptide or the control CLIP peptide for 2 days. (**A**), Representative gating strategy for leukocytes in the spleen. (**B**), Flow cytometry analysis of GluN1 reactive $CD8^+$ and $CD4^+$ T cells in spleen at 7 days and 15 days post immunization based on CD69 and CD25 markers. ANOVA test (* $p < 0.05$; *** $p < 0.001$; **** $p < 0.0001$), n = 5 (D7PI and D15PI).

A.

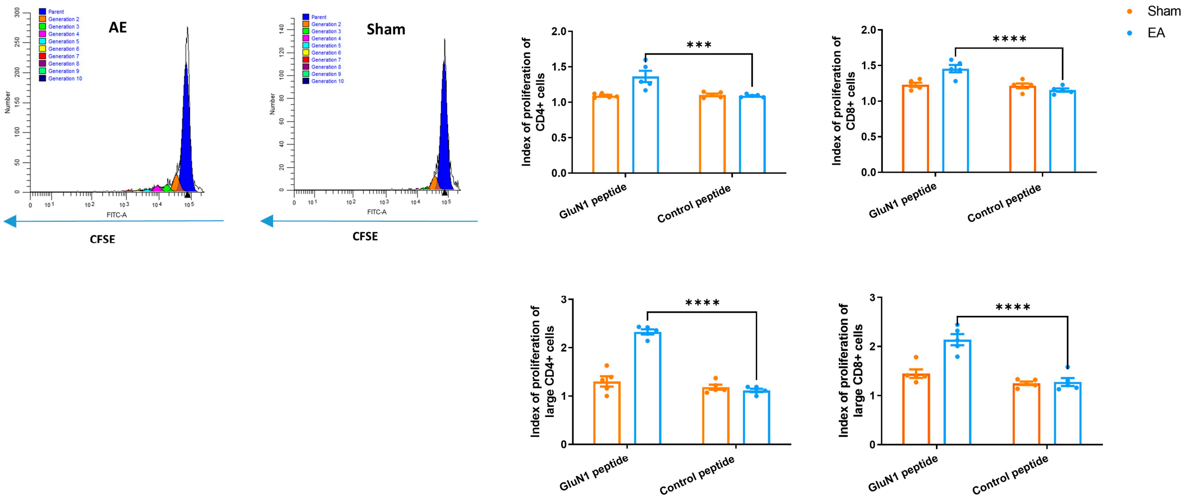

B.

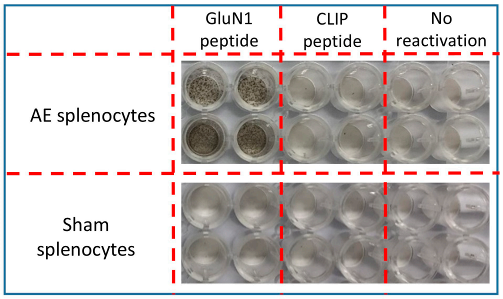

Figure S4. Specificity of the anti-GluN1 T cell in AE mice. (**A**) Flow cytometry analysis of T cell proliferation. Splenocytes from sham or AE mice were marked with CFSE and were cultured with GluN1 peptide or the control CLIP peptide for 4 days. Data were expressed as percentage of parent cells (mean ± SEM), ANOVA test (*** $p < 0.001$; **** $p < 0.0001$), n = 5. (**B**) Elispot analysis. Splenocytes from sham or AE mice were cultured with GluN1 or CLIP peptide for 10 days. IFN-γ production is determined by ELISpot assay after stimulation with GluN1 or CLIP peptide into the wells (n = 2).
